# A Suprainguinal Ultrasound-Guided Fascia Iliaca Plane Injection Using the Anterior Inferior Iliac Spine Landmark: An Anatomy-Based Technical Report

**DOI:** 10.7759/cureus.108979

**Published:** 2026-05-16

**Authors:** Anwar Suhaimi, Teinny Suryadi, Yonghyun Yoon, Sang-Hyun Kim, U-Young Lee, Jihyo Hwang, King Hei Stanley Lam

**Affiliations:** 1 Rehabilitation Medicine, University Malaya Medical Centre, Kuala Lumpur, MYS; 2 Rehabilitation Medicine, University Malaya, Kuala Lumpur, MYS; 3 Physical Medicine and Rehabilitation, Medistra Hospital, Jakarta, IDN; 4 Physical Medicine and Rehabilitation, Synergy Clinic, Jakarta, IDN; 5 Physical Medicine and Rehabilitation, Hermina Podomoro Hospital, Jakarta, IDN; 6 Orthopaedics, International Academy of Musculoskeletal Medicine, Hong Kong, HKG; 7 Orthopaedics, International Academy of Regenerative Medicine, Incheon, KOR; 8 Orthopaedics, MSKUS, San Diego, USA; 9 Orthopaedic Surgery, Hallym University Kangnam Sacred Heart Hospital, Seoul, KOR; 10 Orthopaedic Surgery, Incheon Terminal Orthopedic Surgery Clinic, Incheon, KOR; 11 Anatomy, College of Korean Medicine, Woosuk University, Jeonbuk-do, KOR; 12 Anatomy, Catholic Institute for Applied Anatomy, College of Medicine, Catholic University of Korea, Seoul, KOR; 13 Faculty of Medicine, The Chinese University of Hong Kong, New Territories, HKG; 14 Faculty of Medicine, The University of Hong Kong, Hong Kong, HKG; 15 The Board of Clinical Research, The Hong Kong Institute of Musculoskeletal Medicine, Kowloon, HKG

**Keywords:** chronic pain, fascia iliaca compartment, fascia iliaca plane injection, femoral nerve, hydrodissection, iliacus muscle, interventional pain medicine, lateral femoral cutaneous nerve, suprainguinal fascia iliaca, ultrasonography

## Abstract

The fascia iliaca compartment is a potential interfascial space deep to the fascia iliaca and superficial to the iliacus and psoas muscles. Compared with infrainguinal techniques, the suprainguinal approach is anatomically favorable for cephalad spread beneath the fascia iliaca. Although this approach is well established in perioperative analgesia, detailed procedural descriptions adapted for ultrasound-guided fascial hydrodissection and interventional pain practice remain limited.

This technical report describes an anatomy-based, ultrasound-guided suprainguinal fascia iliaca plane injection using the anterior inferior iliac spine (AIIS) as a practical sonographic landmark. With the patient supine, a high-frequency linear transducer is used to identify the AIIS and is then rotated into an oblique plane directed toward the umbilicus. In this orientation, the iliacus muscle, overlying fascia iliaca, and adjacent abdominal wall musculature can be visualized. The procedural target is the plane between the fascia iliaca and the iliacus muscle. Under in-plane ultrasound guidance, an echogenic needle is advanced from caudad to cephalad into the interfascial plane. Correct needle-tip placement is confirmed with a small test injectate, followed by incremental delivery of the remaining injectate under continuous visualization.

This report emphasizes sono-anatomical orientation, needle trajectory, confirmation of correct fascial plane separation, expected injectate spread, and safety considerations. The manuscript is intended as a technical and educational description rather than as evidence of clinical efficacy. By presenting a reproducible AIIS-based workflow, this report may support clinician training, procedural standardization, and future prospective studies.

## Introduction

The fascia iliaca compartment is a potential space formed by the fascia iliaca overlying the iliacus and psoas musculature. This compartment is of procedural interest because of its relationship to the femoral nerve and lateral femoral cutaneous nerve, with variable continuity toward other branches of the lumbar plexus [1,2]. Modern anatomical work has shown that the fascia iliaca compartment is not simply a uniform cavity, but a structured fibrous space with implications for injectate spread and nerve coverage [2].

Ultrasound-guided fascia iliaca compartment block is widely used in perioperative pain management and, in hip and knee arthroplasty populations, has been associated with reduced postoperative pain and opioid requirements [3]. Among the described approaches, the suprainguinal technique has attracted particular attention because it allows more consistent cranial, or upward, spread beneath the fascia iliaca toward the more proximal course of the target nerves than classic infrainguinal injection [1]. Imaging and cadaveric studies support more reliable spread to the femoral nerve and lateral femoral cutaneous nerve with the suprainguinal approach, whereas obturator nerve involvement remains inconsistent [1,4,5].

Separately, ultrasound-guided hydrodissection has gained interest in interventional pain medicine as a technique in which fluid is injected to separate adjacent tissue layers and create a visible working space under ultrasound guidance [6]. In this context, an interfascial plane refers to the potential space between two fascial or myofascial layers. In peripheral entrapment neuropathies, injectates such as 5% dextrose in water (D5W), saline, local anesthetic, and platelet-rich plasma have been used for perineural or interfascial hydrodissection, although optimal injectate selection remains indication-specific and incompletely defined [7,8]. Dextrose-based injection has also been studied more broadly in chronic musculoskeletal pain, although that literature should not be interpreted as direct evidence for this specific interfascial technique [9].

For image-guided procedures involving the anterior or lateral hip and thigh region, a reproducible landmark-based method for entering the suprainguinal fascia iliaca plane may be useful for both training and future research. In the present technique, the anterior inferior iliac spine (AIIS) was selected as the primary landmark because it is a readily identifiable bony structure on ultrasound and provides a practical starting point for orienting the transducer toward the intended suprainguinal fascia iliaca plane. The purpose of this technical report is therefore to describe a practical ultrasound-guided suprainguinal fascia iliaca plane injection using the AIIS as the principal landmark. The report focuses on sono-anatomical orientation, in-plane needle advancement, confirmation of correct fascial plane separation, anticipated neural coverage, and procedural safety. It is intended as a technical and educational description rather than as evidence of therapeutic efficacy.

## Technical report

Procedural purpose and scope

The objective of the described procedure is to access the suprainguinal fascia iliaca plane in a reproducible manner using the AIIS as a sonographic landmark. Depending on clinical intent, the technique may be used for fascial plane injection, hydrodissection, or local anesthetic-based regional analgesia. The present report is limited to a technical description and anatomical rationale. It does not evaluate comparative effectiveness, long-term outcomes, or superiority over other suprainguinal fascia iliaca techniques.

Patient positioning and equipment

The patient is positioned supine. Mild ipsilateral hip flexion and external rotation may improve comfort and reduce tension in the iliopsoas region; this can be achieved by placing a pillow or bolster beneath the knee. A high-frequency linear transducer (approximately 6-18 MHz) is appropriate in most patients, whereas a lower-frequency curvilinear transducer may be required when the target lies beyond the effective penetration of a linear probe.

A 23-gauge echogenic needle, typically 6-8 cm in length, is preferred for most cases because it provides a practical balance between needle visibility, tactile feedback, and injectate flow. A 25-gauge needle may be chosen in lean patients or when minimizing tissue trauma is prioritized, whereas a 22-gauge needle may be considered when increased shaft stiffness or more efficient delivery of larger injectate volumes is desired. Standard procedural monitoring should be used in accordance with institutional practice, including pulse oximetry and noninvasive blood pressure monitoring when clinically indicated. Resuscitation equipment and, when a local anesthetic is used, readiness to manage local anesthetic systemic toxicity should be ensured.

Sono-anatomical orientation

Step 1: Identification of the AIIS

Scanning begins over the anterior superior iliac spine (ASIS) in a transverse orientation. The transducer is then moved slightly inferiorly and medially until the AIIS is identified as a hyperechoic bony contour with posterior acoustic shadowing. The iliacus muscle is seen deep to the overlying fascial layer and contiguous with the iliac fossa.

Step 2: Oblique AIIS-to-Umbilicus View

From the AIIS, the transducer is rotated into an oblique plane directed toward the umbilicus. This orientation lengthens the visualized suprainguinal iliac fossa and facilitates recognition of the target fascial interface. In this view, the following structures should be identified. 

The AIIS appears as a superficial osseous landmark with posterior acoustic shadowing. The iliacus muscle is visualized as a hypoechoic pennate muscle occupying the iliac fossa. Superficial to the iliacus muscle, the fascia iliaca is seen as a thin hyperechoic line. The overlying abdominal wall musculature, typically the internal oblique and transversus abdominis muscles near their iliac attachment, can also be recognized. When visible, the sartorius muscle may assist with spatial orientation.

The procedural target is the interfascial plane between the fascia iliaca and the superficial surface of the iliacus muscle. 

**Figure 1:**
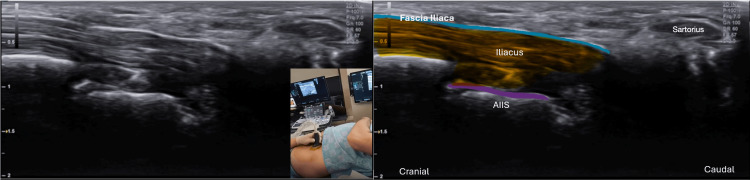
Ultrasound transducer positioning and sono-anatomy of the suprainguinal fascia iliaca plane. The patient is positioned supine with the ipsilateral hip slightly flexed and externally rotated. A high-frequency linear transducer is placed in an oblique longitudinal plane aligned from the anterior inferior iliac spine (AIIS) toward the umbilicus. The corresponding ultrasound image shows the AIIS (purple line) as a hyperechoic bony contour with posterior acoustic shadowing. The iliacus muscle (brown) fills the iliac fossa, and the fascia iliaca (blue line) is visualized as a hyperechoic line superficial to the iliacus. The sartorius muscle is also identified. This orientation establishes the target plane for hydrodissection. The images were derived originally from ultrasound images taken by Dr Yonghyun Yoon, Dr Teinny Suryadi, and Prof King Hei Stanley Lam and subsequently edited using the Procreate app (Hobart, Australia) on an iPad (Apple Inc., Cupertino, CA) by Dr Teinny Suryadi and Prof King Hei Stanley Lam.

The AIIS-to-umbilicus transducer orientation and the relevant sono-anatomical landmarks are shown in Figure 1.

Needle insertion and hydrodissection

After skin antisepsis and sterile preparation, the needle is inserted in plane with the transducer from the caudad end of the probe and advanced in a caudad-to-cephalad direction toward the fascia iliaca plane. Continuous visualization of the needle shaft and tip is essential. Minor adjustments in probe tilt, heel-toe maneuver, or needle angle may be required to optimize needle conspicuity.

When the needle tip reaches the fascia iliaca, a subtle change in tissue resistance may be appreciated. However, tactile sensation alone should not be relied upon. Correct placement should be confirmed sonographically by injection of a small test volume, typically 1-2 mL of sterile saline, D5W, or other chosen injectate. Proper placement is indicated by visible separation of the hyperechoic fascia iliaca from the iliacus muscle, creating an anechoic or hypoechoic cleft along the interfascial plane.

**Figure 2:**
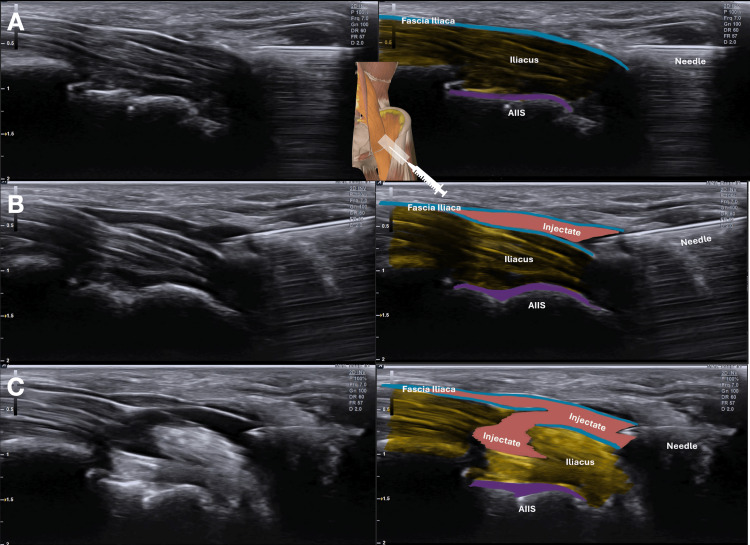
In-plane needle trajectory and hydrodissection of the fascia iliaca plane. (A) A 23-gauge, 6 cm echogenic needle is advanced in-plane from caudad to cephalad, targeting the interface between the fascia iliaca (blue line) and the iliacus muscle (brown). The anterior inferior iliac spine (AIIS) (purple line) serves as the primary bony landmark. (B) Following needle-tip confirmation, a test bolus (1-2 mL) is injected, demonstrating partial hydrodissection as the injectate (pink) begins to separate the fascia iliaca from the underlying iliacus. (C) Complete hydrodissection is achieved after incremental delivery of the planned injectate volume, with the fascial plane fully expanded. The corresponding real-time video is available (Video 1). The images were derived originally from ultrasound images taken by Dr Yonghyun Yoon, Dr Teinny Suryadi, and Prof King Hei Stanley Lam and subsequently edited using the Procreate app (Hobart, Australia) on an iPad (Apple Inc., Cupertino, CA) by Dr Teinny Suryadi and Prof King Hei Stanley Lam.

The in-plane needle trajectory and the sequential appearance of fascial plane opening during hydrodissection are demonstrated in Figure 2.

A representative real-time ultrasound clip of the described technique is provided in Video 1.

**Video 1:** Real-time ultrasound-guided suprainguinal fascia iliaca plane injection using the AIIS landmark. Real-time ultrasound video demonstrating the suprainguinal fascia iliaca plane injection technique. An echogenic needle is advanced in-plane from caudad to cephalad toward the plane between the fascia iliaca and the iliacus muscle. A small test bolus confirms correct needle-tip placement, followed by incremental delivery of the remaining injectate to facilitate cephalad fascial plane spread toward the psoas region. AIIS: anterior inferior iliac spine The video is taken by Dr Teinny Suryadi, Dr Yonghyun Yoon, and Prof King Hei Stanley Lam.

**Figure 3:**
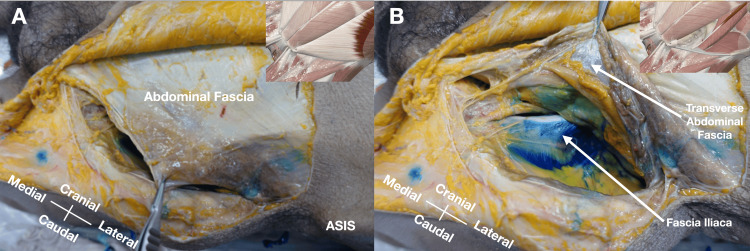
Illustrative cadaveric demonstration of injectate spread following AIIS-guided injection. (A) External view following injection of blue dye using the described suprainguinal technique. The injection site is identified as a blue-stained point in the infrainguinal region after reflection of the skin and subcutaneous tissue. (B) Deeper dissection with the abdominal wall muscles reflected exposes the iliacus muscle overlying the AIIS. Blue dye staining is evident within the fascia iliaca plane, confirming deposition and early spread along the intended interfascial compartment. AIIS: anterior inferior iliac spine The images were taken by Dr Yonghyun Yoon and Prof King Hei Stanley Lam.

**Figure 4:**
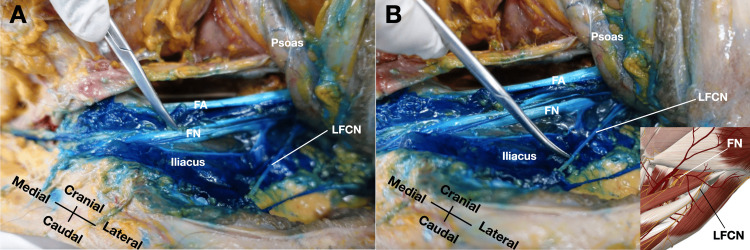
Staining of the femoral and lateral femoral cutaneous nerves following AIIS-guided injection. With the abdominal wall muscles and psoas retracted, (A) the femoral nerve (FN) is clearly stained by the dye adjacent to the femoral artery (FA), with a metal instrument indicating its course. (B) The lateral femoral cutaneous nerve (LFCN) is similarly stained, consistent with the expected anatomical reach of injectate delivered within the described plane. The images were taken by Dr YongHyun Yoon and Prof King Hei Stanley Lam.

Representative cadaveric images illustrating dye spread within the fascia iliaca plane and staining of the femoral and lateral femoral cutaneous nerves are shown in Figure 3 and Figure 4. Representative cadaveric images were obtained after ultrasound-guided injection of 10 mL of methylene blue in four cadaveric specimens (eight sides), followed by dissection to assess gross spread within the fascia iliaca plane.

If injectate instead disperses within muscle fibers or fails to open a linear plane, the tip is likely intramuscular or otherwise malpositioned. In that circumstance, the needle should be withdrawn slightly and redirected until smooth plane separation is obtained.

Once correct placement is confirmed, the remainder of the injectate is delivered incrementally under continuous ultrasound observation. The exact composition and volume depend on procedural intent. For hydrodissection-oriented applications, a larger volume may be selected to promote longitudinal plane opening and cephalad spread; however, the optimal injectate and volume for chronic pain indications remain undefined [6-8]. When a local anesthetic is used, dose calculation must be individualized and cumulative toxicity limits strictly observed.

The desired sonographic endpoint is expansion of the fascia iliaca plane with spread tracking cephalad along the iliacus surface toward the psoas region. Needle advancement within the already-opened plane may be performed cautiously, if required, to facilitate more proximal spread.

Procedural pearls and pitfalls

Several practical points may improve reproducibility. The AIIS is a useful starting landmark because it is typically easier to identify consistently than the intended suprainguinal fascial target itself. The most important technical confirmation is not a subjective “pop,” but direct visualization of fascial plane separation. A shallowly advancing needle often improves in-plane visualization compared with a steep trajectory. Apparent injectate spread without linear fascial separation should prompt reassessment for intramuscular needle placement. In patients with deeper targets or reduced image quality, a curvilinear probe may provide better penetration at the cost of fine near-field resolution.

Safety considerations

Although the described plane is superficial relative to the pelvis, the procedure should be performed with standard ultrasound-guided injection precautions. Potential complications include inadvertent intramuscular injection, vascular puncture, infection, transient sensory change, and, if a local anesthetic is administered, local anesthetic systemic toxicity and quadriceps weakness. Because femoral nerve involvement may impair knee extension, patients should be reassessed for safe ambulation before discharge when motor-blocking injectates are used. Additional caution is warranted in anticoagulated patients or in those with altered anatomy from prior surgery, trauma, or substantial soft tissue distortion.

Post-procedure assessment

Following injection, the patient should be observed in accordance with the injectate used and the clinical setting. If a local anesthetic has been administered, sensory changes in the anterior thigh and lateral thigh may be documented, and quadriceps strength should be checked before ambulation. If the procedure is performed for an interventional pain indication, baseline and immediate post-procedure pain scores may be recorded for clinical documentation; however, such observations should not be interpreted as evidence of durable efficacy in the absence of structured follow-up data.

## Discussion

This technical report describes a practical ultrasound-guided method for entering the suprainguinal fascia iliaca plane using the AIIS as the principal landmark. The report is intended to provide an anatomy-based workflow that may be useful for procedural teaching, standardization, and future study.

The anatomical basis for this approach is supported by prior work on the fascia iliaca compartment and its injectate spread characteristics. Prior studies have shown that a suprainguinal injection can produce more consistent spread toward the anatomical location of the target nerves than an infrainguinal approach [1]. A complementary anatomical study has demonstrated that the fascia iliaca compartment is defined by a fibrous architecture that may influence the directional behavior of injectate [2]. More recent cadaveric and radiological work has emphasized that, although the suprainguinal approach improves cranial spread, obturator nerve involvement remains unreliable even with larger-volume injection [4,5]. From a procedural standpoint, this is highly relevant: the suprainguinal fascia iliaca plane should not be assumed to provide consistent obturator coverage.

For that reason, the likely neural targets of this technique should be described conservatively. Based on existing anatomical and imaging literature, the femoral nerve and lateral femoral cutaneous nerve are the structures most likely to be reached by injectate in this plane [1,4,5]. This is relevant to image-guided interventions involving the anterior and lateral thigh or selected anterior hip pain patterns, while acknowledging that hip joint innervation is complex and often multimodal [10]. The technique should therefore be understood as one potential component of regional analgesia or image-guided interfascial intervention, rather than a comprehensive solution for all pain arising from the hip or knee.

The present report also differs conceptually from conventional perioperative fascia iliaca block literature. In perioperative settings, fascia iliaca injection is typically performed to achieve sensory analgesia with local anesthetic [3,11]. In contrast, some interventional pain practitioners may seek deliberate hydrodissection of the fascial interface using non-anesthetic or low-anesthetic injectates to obtain mechanical plane separation and improved tissue glide [6-8]. That rationale is plausible and is supported indirectly by the broader hydrodissection literature, particularly in peripheral entrapment neuropathies [6-9]. However, this body of evidence does not directly validate the present suprainguinal fascia iliaca plane technique, and the current report should not be interpreted as establishing therapeutic equivalence. Nor does the current report establish that hydrodissection of the suprainguinal fascia iliaca plane improves chronic pain outcomes or define the optimal injectate composition, total volume, or retreatment interval for any chronic pain indication.

The AIIS-based orientation may nevertheless offer practical advantages. First, it begins with a familiar and readily visualized bony landmark. Second, rotating from the AIIS toward the umbilicus provides a reproducible method of aligning the transducer with the intended suprainguinal plane. Third, this view facilitates dynamic confirmation that the fascia iliaca is being separated from the iliacus rather than unintentionally entering the muscle. These features may be especially useful for clinicians transitioning from more familiar infrainguinal scanning approaches to a suprainguinal target.

This report has several limitations. First, it is a technical description and not an outcomes study. No conclusions can be drawn regarding analgesic efficacy, duration of benefit, patient selection, or comparative effectiveness. Second, any accompanying cadaveric images should be interpreted as anatomical illustrations rather than definitive proof of reproducible in vivo spread, unless supported by formally reported specimen methods and results. The cadaveric component included only four specimens (eight sides), which is insufficient to characterize broader anatomical variability; accordingly, these findings should be interpreted as illustrative rather than representative. Third, the relationship between volume and neural coverage in hydrodissection-oriented practice remains uncertain. Although larger volumes may facilitate longitudinal spread, volume should not be equated with predictable block completeness, particularly with respect to the obturator nerve [4,5]. Fourth, the safety profile of this approach in chronic pain practice has not been systematically studied.

Prospective research is needed to define procedural indications, reproducibility across operators, optimal injectate selection, dose-volume relationships, safety outcomes, and patient-reported clinical benefit. Studies incorporating structured follow-up and, where feasible, post-procedural imaging could help clarify whether the anatomical promise of this approach translates into meaningful clinical value in chronic pain populations.

## Conclusions

This technical report describes an AIIS-based ultrasound-guided method for accessing the suprainguinal fascia iliaca plane. The technique emphasizes reproducible sonographic orientation, in-plane needle guidance, direct confirmation of fascial plane separation, and realistic expectations regarding neural coverage. Based on existing anatomical and imaging literature, the approach is most likely to affect the femoral and lateral femoral cutaneous nerves, whereas obturator nerve involvement should not be presumed. The report is intended as an educational and procedural resource. Clinical effectiveness, optimal injectate strategy, and safety in chronic pain populations require prospective investigation.
